# Editorial: How animals affect us: examining the influence of human-animal interactions on human's health

**DOI:** 10.3389/fvets.2024.1509960

**Published:** 2024-11-08

**Authors:** Fernando Capela e Silva, Emily Kieson, Alexandra N. Stergiou, Inês Pereira-Figueiredo

**Affiliations:** ^1^Department of Medical and Health Sciences, School of Health and Human Development, MED – Mediterranean Institute for Agriculture, Environment and Development and CHANGE – Global Change and Sustainability Institute, Institute for Advanced Studies and Research, University of Évora, Evora, Portugal; ^2^Equine International Corp., Boston, MA, United States; ^3^Department of Early Years Learning and Care, University of Ioannina, Ioannina, Greece; ^4^TheKidsFellows, Research Group in Anthrozoology, Idanha-a-Nova, Portugal

**Keywords:** anthrozoology, Human-Animal Interactions (HAI), Animal Assisted Interventions (AAI), Animal Assisted Therapy/Activity (AAT/A), human health

## Introduction

In the history of human societies, the most important and decisive stage was certainly the transition from the appropriation of spontaneous resources to the production of domesticated resources. Animal domestication definition is not unique and can be based on several criteria and thought of in several pathways (1–3). Through domestication man learned to take advantage of the potential of the animal that best suited his needs, which allowed us to understand that domestic animals are not the result (only) of natural selection but rather of so-called artificial selection (4). Practiced over thousands of years, artificial selection gave rise to the large number of breeds of different domesticated species that are currently known, mostly reflecting the different ways of using animals according to different regions (5, 6).

The first domesticated species was the dog (7), probably domesticated by hunters, followed later by the most economically important species—sheep, goats, cattle and pigs,—for their primary products, meat and leathers. Later, appeared horses, used mainly to transport people and goods/products and for traction, a phase that corresponds to a period in which man began to exploit animals for their secondary products, such as strength, milk, wool and manure (5).

Certain behavioral and temperament characteristics, namely ease of environmental adaptation, tendency to escape or aggression, tameness, docility, sociability, emotional reactions toward humans, have made certain animal taxa, and certain individuals within taxa, better candidates for domestication and a lasting relationship with humans (1). In addition to these interactions, more of a material and instrumental nature, others emerged from an affective point of view, giving rise to animals kept as pets including the aforementioned and newly domesticated mammals and birds as well as species of wild mammals, birds, fishes, reptiles and arthropods (1).

These kind of Human-Animal Interactions (HAI) of an affective nature have developed and consolidated (8) to such an extent that some species are currently used as Animal Assisted Therapy (AAT) in humans, in certain situations, and with great potential for healing, or at least alleviating and improving their symptoms. AAT are practices that incorporate HAI in a structured and goal-oriented manner into patient treatment plans for the benefit of their health (9). On the other hand, Animal Assisted Activities (AAA), another type of HAI, can be performed by HAI professionals or volunteers in a variety of settings to improve participants' quality of life, with or without consideration of health-related goals (9). It is also known that the bonds between humans and animals are mutually beneficial relationships for people, as well as for the animals involved, so in this context health is seen from a broad perspective, including not only physical benefits, but also mental, emotional and social wellbeing. In other words, domestication, which initially had an essentially “utilitarian” character, always aiming at a specific objective (source of food, protection, etc.), gradually acquired a new facet linked to the affective aspect, thus giving rise to pets, which today represent a decisive factor in combating one of the main problems of contemporary society: loneliness. In this context, animals represent companionship and affection, especially for the elderly (10) and children (11–13), with enormous physical and emotional benefits. Several scientific studies have proven a positive relationship between living with an animal and a reduction in cases of depression and suicide rates, improved blood pressure control, etc. Thus, the HAI/AAA/Animal Assisted Interventions (AAI) are a growing, multifaceted and multidisciplinary sector made up of industries and complementary professionals who work alongside animals as a fundamental part of their service and interventions (14, 15). AAI professionals use the inclusion of animals in their services to obtain therapeutic gains and improvements in the health and wellbeing of their clients, and of the animals themselves. Their approaches are diverse, including animal-assisted therapies in families, hospitals, institutions and organizations created for this purpose, in sports and at work, with vulnerable populations, etc. All these kinds of interactions/interventions have been widely used with excellent results, using the most diverse animal species, namely horses, dogs, cats, dolphins, or through simple contact with nature and its plant and animal biodiversity. However, from whatever perspective we consider interactions/interventions, we must always do so based on ethical principles and with respect for their wellbeing (16, 17).

*Frontiers* has given extensive emphasis to this topic, with several Research Topics having been published in recent years, which we will cite when deemed appropriate. Included in this *Research Topic*, whose aim was to contribute to a better understanding of the healing power of the bond between humans and animals and how these interactions affect each other, are 12 articles: 8 Original research, 1 Systematic review, 1 Mini review, 1 Brief research report, and 1 Perspective. However, we will present and explain these articles, not by their typology, but by subjects/species in which there is something in common between them, covering a wide variety of themes, appropriately identified.

## Dogs and cats

The first domesticated species was the dog (*Canis familiaris*) and, given its propensity to form secure and strong bonds with humans, it consolidated a privileged status as a companion animal and was used as a working animal in various activities, including hunting, rescue, military, man tracking, sled dogs, cinema, etc (18–25). On the other hand, dogs are considered the most important species in AAT, and there are several benefits linked to their involvement in different therapeutic areas (19, 26).

Although in today's modern societies, both dogs and cats have similar roles as pets in the lives of humans, there are some important differences between the two species (27), making the cat an exception to generalizations of development and settlement of animal-human interactions (5). The cat (*Felis catus*) is a relatively solitary species, individuals interact very little with each other, with minimal contact, except during the reproductive period, and they are very fierce in relation to the territory, maintaining a strong connection to it (5, 27). A domestic cat is more linked to the home occupied by its owners/guardians than to human beings themselves (5) and this behavior and other behavioral characteristics (28) may partly explain its lower use in HAI/AAA/AAI, compared to dogs. Even so, domestic cats are immensely popular companion animals in households around the world, in the USA (28, 29) and in the European Union, where the population of pet cats is estimated to be 113 million (29).

In this Research Topic, four manuscripts on therapies using dogs/cats were published. Late adolescence is a crucial period of individual development and growth, in which new relationships are established, and in which involvement with family and the local community contributes to wellbeing, playing a significant role in the formation of personality. On the other hand, pets, such as dogs and cats, also increase interactions with family and the local community, thus contributing to increased wellbeing in adolescents. Using questionnaires administered to high school and college students, Koyasu et al. assessed the effects of the experience of having pets on involvement with family and the local community, wellbeing and general confidence. The results obtained revealed that girls who had dogs or cats had greater wellbeing and general confidence through involvement with their families, but this did not happen with boys. The authors suggest future cohort studies examining the effects of pets in each age group.

Some studies evaluating the association between pets and cardiovascular disease have produced inconsistent results, which may be explained, at least in part, by variations in age and sex among study populations. The study by Watson et al. included 6,632 American Gut Project participants who are US residents ≥40 years old. The authors, using multivariable adjusted logistic regression, estimated the association between pet ownership and the risk of cardiovascular disease and assessed the effect of age and sex. The results indicated that cat ownership, but not dog, is significantly associated with a lower risk of cardiovascular disease. Conversely, significant interactions were observed between cat and dog ownership with age but not sex, indicating that cardiovascular risk varies by the combination of age and pet ownership. This study emphasizes the importance of pets in human cardiovascular health, suggesting that the ideal choice of pets depends on age, although, according to the authors, more studies are needed to assess causality.

Although interacting with animals has been shown to have benefits for human health, there are limitations to physical interaction for safety reasons due to zoonosis risks, including COVID-19 (30). As an alternative, Na and Dong created three types of human-animal interaction (HAI) content based on mixed reality (MR) and experimentally evaluated their effect on reducing mental stress: observation of the movement of a non-reactive virtual cat, interaction with a virtual cat whose responses can be viewed and interaction with a virtual cat whose responses can be viewed or heard. Thirty healthy young women underwent a mental arithmetic task in order to induce mild mental stress before experiencing each content, and during the experiment the electrocardiogram was recorded continuously, and the psychological state was assessed using a questionnaire. The results showed that MRI-based virtual cat content significantly reduced mental stress and induced positive emotions after stressful situations, in particular, when the virtual cat provided audiovisual feedback. Based on the results obtained, the authors suggest that this method should be investigated further to see if it can replace real HAI in the management of human mental health.

Aging is a continuous process of natural changes, during which many physiological functions begin to gradually decline, including decline in cognitive function (memory, learning, perception, consciousness) and risk of frailty and comorbidities that may require careful nutritional planning (31). Organ failure is the leading incurable, life-limiting condition of older dogs and cats, many suffering from the same illnesses as humans (32). On the other hand, improvements in veterinary healthcare in recent decades, through advances in diagnostics and treatments, mean that we now have a significant population of small geriatric companion animals. This necessarily means that owners increasingly face the challenges of caring for elderly pets with life-limiting illnesses and making end-of-life decisions. In the article by Lam et al. its two first authors, based on their own experience, present their personal perspective and what they consider to be the veterinarian's perspective on these issues. In order to improve the quality of life of both animals and their owners, they suggest that three communication elements are primary, namely: empathic communication and shared decision-making; managing progressive symptoms, and; advanced directives.

## Horses

Although the horse (*Equus caballus*) was domesticated after dogs, cattle, pigs, sheep, and goats, it represents the domestic animal that had the most impact on the development of human civilization, providing rapid transportation, considerably altering the speed and magnitude of the circulation of goods and people, revolutionizing war and agriculture, and profoundly influencing the political-economic trajectory of human societies (33, 34). There are currently around 60 million horses on the planet which are mainly confined to the sports and leisure industries in most developed countries, although in developing countries they fulfill their traditional roles, providing transport, draft power, meat, milk, hair and leather (34). Horses are also a species widely used and important in animal-assisted therapies, which can be divided into two broad categories: therapy aimed at improving mental health and therapy aimed at improving physical health (35–37).

In this Research Topic, five manuscripts on therapies using horses were published. Smith et al. used qualitative methods to assess the experiences of owners and veterinarians of older horses, considering that the importance of veterinarian-owner interactions in establishing future veterinary care needs may be underestimated. Analysis of the data, collected between 2019 and 2022, identified that owners carried out an ongoing and interactive process of assessment, monitoring and decision-making in relation to the animal and any observed changes. The results obtained demonstrate how issues of health, disease and the role of professionalized forms of medical knowledge are not static, but constantly change, interacting over time.

Stergiou et al. evaluated the efficacy of Equine Assisted Therapy (EAT) in children with Cerebral Palsy, in terms of gross motor function, performance, and spasticity as well as whether this improvement can be maintained for 2 months after the end of the intervention. Five measurements were considered: Gross Motor Function Measure-88 (GMFM-88), Gross Motor Performance Measure (GMPM), Gross Motor Function Classification System (GMFCS), Modified Ashworth Scale, Wechsler Intelligence Scale for Children and statistically significant improvements were achieved for some children in Gross Motor Function Measure and all its subcategories (which remained consistent for 2 months after the last session of the intervention), also in total Gross Motor Performance Measure and all subcategories. According to the observed results the authors conclude that EAT improves motor ability (qualitatively and quantitatively) in children with Cerebral Palsy, with clinical significance in gross motor function.

Mattila-Rautiainen et al. evaluated the impact of Equine Facilitated Therapy (EFT) on perceived physical performance, pain level, pain acceptance, depression and anxiety, and quality of life in a 12-week intervention in a sample of 22 patients with chronic low back pain (LBP). Participants received EFT, supervised by physiotherapists, combining quantitative and qualitative methods, and data were collected through questionnaires, interviews, and patient data repositories. Horse welfare was taken into account in the basic training and in the research environment. The results of this work indicate a significant increase in satisfaction levels and a decline in the amount of perceived depression, and only two of the 22 participants returned with recurrent symptoms after 6 months to the pain clinic. The participant interviews revealed three important domains of experience during coding: physical-, psychological-, and social that link to the research question and suggest impact for the recovery from the human-animal interaction.

Rigby reviews the effect that continued and repeated stress can have on the rider during equine-assisted services, evaluating different neuroendocrine biomarkers, namely immunoglobulin A, serotonin, cortisol, progesterone and oxytocin. The results are mixed regarding the effects of these hormones on the rider's physiology before, during and after equine-assisted services. The author suggests that future work should adopt an interdisciplinary approach, with properly controlled studies, appropriate treatments and experimental rigor, considering exogenous and endogenous factors that influence the rider's physiology.

Adaptive or therapeutic riding (A/TR) is a recreational activity that provides mounted and ground-based horsemanship opportunities adapted to the abilities of the participants and providing physical and psychological benefits to participants with diverse disabilities, promoting higher quality of life. The aim of the study of Hanson et al. was to identify whether, and how, professionals in A/TR, standardize the assessment of participant outcomes, and the advantages and barriers to this standardization. According to survey results, assessments are not standardized, although the A/TR professionals believe that their establishment would strengthen the profession, obtain funding, and communicate about A/TR services to a broad audience, aspects relevant to all age groups and populations that use these services. Respondents identified several barriers to implementing standardized assessments, including time, system and expertise constraints, cost, time required, and not being available in article or computer format. The authors conclude that standardized assessments can be a strong support for the A/TR profession, although they must meet the unique needs of these professionals.

## Animal-assisted interventions and education programs

Educational programs and courses on animal-assisted interventions (AAI), their ethical and practical principles, and models and theories that support the psychophysiological effects of human-animal interactions (HAI), are being implemented worldwide.

A growing number of studies in the field of Social Work (SW) address incorporating the presence of animals into the practice and understanding of social support and therapeutic components. Rusu and Davis present a step-by-step approach to including HAI knowledge and practice in teaching Yalom principles and therapeutic factors in group therapy to SW students. Based on a qualitative analysis of existing literature and the results of several research projects in the area of HAI, the authors propose a strategy to include examples and research-based theories that support the beneficial effects of HAI toward interdisciplinary understanding of the primary factors of Yalom in the therapeutic process, such as: instillation of hope, corrective recapitulation of the primary family group, development of socialization techniques, imitative behavior, interpersonal learning, and group cohesion. For each of these factors, the authors discuss the applied values of HAI, emphasizing the added value of the presence of animals in group therapy environments from the perspective of the dynamics of interspecific social networks. Based on the results of their qualitative analysis, the authors recommend that Yalom's group therapy factors be fostered in AAI-group providing information/psychoeducation to professionals and group participants about the theories that support the beneficial effects of direct contact with animals and about the potential mechanisms of positive interspecific interactions.

In the United States of America, a growing number of higher education institutions offer courses on adaptive/therapeutic riding and the incorporation of horses into human services areas, namely education, psychotherapy, occupational therapy, physical therapy, and speech-language pathology. Since the first study to identify courses in these areas was published in 2018, Connolly and Ekholm Fry publish a scoping review to evaluate the evolution, in these types of institutions, of courses on the use of horses in human services. The authors identified 122 courses offered by 48 higher education institutions in 29 states in the following areas: adaptive/therapeutic riding, mental health, education/learning, and equine movement in physical therapy, occupational therapy, and speech-language pathology (hippotherapy), most of which offered at both the undergraduate and graduate levels. The results suggest avenues for reflection and discussion about changes over time, as well as challenges and opportunities for academic programs of courses on the use of horses in human services.

## Wildlife immersion experiences

The health benefits of contact with nature, particularly mental health, have long been recognized (38), especially in urban populations where people are exposed to excessive stimuli such as noise, light pollution, time pressure and a fast pace of life (39). The relaxing potential of immersion in nature appears to be an essential protective factor, through slow walks through a forest landscape, and the enjoyment of odorants generated by nature (40), nature sounds (41, 42), nature-based mindfulness and therapeutic ornithology (43). The results of Simonienko et al. (43), indicated that forest therapy, nature-based mindfulness and therapeutic ornithology act differently, although they have many common characteristics, so they can work as an effective combination to deal with different types of stress and anxiety symptoms. The results of Peterson et al. (44) show that even half an hour of birdwatching can make us happier, healthier and help foster a deeper connection with nature. On the other hand, listening to birdsong was linked to perceptions of lower stress and attention recovery (45). In this way, visits to bird-rich habitats can become part of social prescribing protocols, playing an important role in preventing mental health problems, complementing interventions (46).

In the manuscript included in this Research Topic, Perry et al. explore the potential of Animal Assisted Interventions (AAI) in improving the physical and psychological health of veterans with post-traumatic stress disorder (PTSD). To this end, they followed, for an average of 15.1 weeks, 19 veterans with PTSD/PTSD symptoms through a series of 8 immersion experiences in nature and wildlife to assess their feasibility and effectiveness. The intervention comprised an initial forest walk, assisting with wildlife rehabilitation, observation in a wildlife sanctuary and bird watching. This AAI nature/wildlife immersion intervention proved to be viable, acceptable and safe, with participants expressing pleasure and satisfaction with the activities, while also highlighting their concern for the welfare of the animals. The authors conclude that these wildlife AAI immersion experiences are feasible and can be safely administered to veterans with PTSD/PTSD symptoms.

## In summary

Human-animal bonds are mutually beneficial relationships that hold the potential to nurture One Health for People as well as the animals involved. Health in this context includes not only the physical benefit, but the mental, emotional, and social wellbeing of both people and animals. Animal-Assisted Interventions (AAI) is a growing, multi-faceted and multi-disciplinary sector comprised of complementary industries and professionals working alongside animals as a key part of their service. The goal of this Research Topic is to contribute to a better understanding of the Healing power of connecting with animals and how the interactions involving humans and animal affect each other.
